# Association of LIPC -250G/A and -514C/T polymorphisms and hypertension: a systematic review and meta-analysis

**DOI:** 10.1186/s12944-018-0884-4

**Published:** 2018-10-15

**Authors:** Xingsheng Zhao, Yu Ren, Hui Li, Yun Wu

**Affiliations:** 1 Cardiology Department, Inner Mongolia People’s Hospital, Zhao Wuda Road, Saipan District, Hohhot, 010017 Inner Mongolia Autonomous Region China; 2 Clinical Medical Research Center, Inner Mongolia People’s Hospital, Hohhot, 010017 Inner Mongolia Autonomous Region China

**Keywords:** LIPC rs2070895 polymorphism, LIPC rs1800588 polymorphism, Hypertension, Hepatic lipase C, Genetic models

## Abstract

**Background:**

Hypertension is the most common chronic disease, and most important risk factor for cardiovascular disease. This meta-analysis aimed to explore the association between hepatic lipase gene (LIPC) gene -250G/A (rs2070895) and -514C/T (rs1800588) polymorphisms and the susceptibility to hypertension.

**Methods:**

Published studies were searched using the PubMed, Embase and Cochrane Library databases. Newcastle-Ottawa Scale (NOS) was used to assess the quality of the included studies. Sensitivity analysis was performed using “leave one out” method. Egger’s test was used to evaluate the publication bias. The random effect model was used to calculate the pooled effect size if *P* < 0.05 or I^2^ ≥ 50%; otherwise, the fixed effect model was selected.

**Results:**

Four eligible studies, including 2599 participants, were enrolled in the included studies from 2007 to 2014. Quality evaluation revealed that each study had high NOS scores ranged from 5 to 7. The LIPC rs1800588 polymorphism was not found to be associated with the susceptibility to hypertension under all genetic models (T vs C, *P* = 0.38; CT vs CC, *P* = 0.46; TT vs CC, *P* = 0.38; TT vs CC + CT, *P* = 0.54; TT + CT vs CC, *P* = 0.34). Notably, the frequencies of the AA+GA genotypes of LIPC rs2070895 polymorphism were related to an increased risk of hypertension (AA+GA vs. GG, OR = 1.1954, 95% CI: 1.0001–1.4288, *P* = 0.05).

**Conclusion:**

The LIPC rs2070895 polymorphism was found to be related to an increased risk of hypertension. However, LIPC rs1800588 polymorphism was not associated with the susceptibility to hypertension.

## Highlight

1. The LIPC rs2070895 polymorphism was related to susceptibility with hypertension.
2. The LIPC rs1800588 polymorphism was not strongly associated with the increased risk of hypertension.

## Background

The main characteristic of hypertension [1–3] is increased arterial blood pressure including systolic and/or diastolic blood pressure [4, 5]; wherein the hypertension is diagnosed as systolic blood pressure higher than 140 mmHg or diastolic blood pressure higher than 90 mmHg, accompanied by the clinical syndrome of the function or organic damage to the heart, brain, kidney and other organs. Hypertension is the most common chronic disease [6] and an important risk factor for cardiovascular disease.

Hepatic lipase (LIPC), a member of the lipase family, is located on the long arm of chromosome 15 (15 q21) and has a total length of 35 kb, comprising nine exons and eight introns [7–10]. LIPC encodes hepatic triglyceride lipase, which is an extracellular protein synthesized by liver parenchyma cells and plays a critical role in lipoprotein metabolism. Hepatic lipase serves an important role inremodeling of low-density liporprotein, high-density lipoprotein, remnant, and the production of small, dense low density lipoprotein [11]. A previous study reported that the lipids and lipoprotein concentrations in blood serum are significantly correlated with primary hypertension and hyperhomocysteinemia [12]. Additionally, the triglycerides and the triglyceride to high-density liproprotein cholesterol ratio are strongly correlated with the incidence hypertension in Middle Eastern women [13]. These findings indicate that disruptions of hepatic lipase is a risk factor for hypertension. The -514C/T (rs1800588) and -250G/A (rs2070895) single nucleotide polymorphisms (SNPs) are two common variants in the promoter region of LIPC and both present obvious chain imbalance [7, 8]. LIPC mutation may be related to the occurrence of a variety of diseases. The -514 T allele of LIPC is associated with the occurrence of insulin resistance syndrome and type 2 diabetes [14]. The LIPC-250G/A loci polymorphism is related to insulin resistance and dyslipidemia [15]. Recent studies showed that mutations in the liver lipase gene are associated with coronary heart disease and cerebral infarction [16, 17]. The relationships between LIPC polymorphisms and metabolic diseases have been widely examined.

Hypertension is an important risk factor for cardiovascular disease including coronary heart disease and cerebral infarction, among others. An increasing number of studies has focused on LIPC gene and hypertension. However, many of these studies included small sample size, and showed controversial results. Thus, this meta-analysis was performed to pool the studies of the frequency distribution of LIPC rs2070895 and rs1800588 polymorphism in hypertension patients and normal individuals to determine the correlation between LIPC gene polymorphism and hypertension, as well as to provide information for guiding future clinical studies.

## Methods

### Search strategy

Relevant studies were searched using the PubMed, Embase and Cochrane Library databases. The following search keywords were used: (“Hypertension” OR “hypertensive” OR “High Blood Pressure”) AND (“LIPC” OR “hepatic lipase gene” OR “rs2070895” OR “-250G/A” OR “-514C/T” OR “C-480 T” OR “rs1800588”) AND (“polymorphic*” OR “genetic” OR “variant”). The search was performed by two reviewers. The retrieval deadline was May 1, 2018 with no language restrictions. This study followed the analysis criteria of Preferred Reporting Items for Systematic Reviews and Meta-Analyses and the participants, interventions, comparison and outcome measures principle.

### Inclusion and exclusion criteria

The included studies conformed to the following criteria: (1) the studies examined the frequency distribution of LIPC -250G/A and -514C/T polymorphisms in hypertension and non-hypertension patients; (2) the studies reported precise genotype or allele frequency data in both groups; (3) the study was a case-control analysis.

Additionally, the studies were excluded if: (1) the data were incomplete, and thus could not be statistically analyzed; (2) the studies were non-treatise literatures, such as reviews, letters, or comments; (3) the study was a repetitive publication or based on data used for multiple studies. In this case, only the most recent study or that with the most complete information was included.

### Data extraction and quality evaluation

Two reviewers independently extracted relevant data from the included publications. The following information was extracted: name of first author, published year, study regions, detection method of genetic polymorphism, number of participants in hypertension and non-hypertension groups, number of each genotype with LIPC (rs2070895 and rs1800588) polymorphisms in both groups, and demographic characters including age and gender. The quality of each included study was evaluated based on the Newcastle-Ottawa Scale (NOS) recommended by the Agency for Healthcare Research and Quality of the US [18]. Disputes were resolved by discussion with a third authorduring data extraction and quality evaluation.

### Statistical analysis

Hardy-Weinberg equilibrium (HWE) test was performed based on the χ^2^ test to determine the genotype stability of LIPC polymorphisms in the control group [19]. R 3.12 was used to conduct the meta-analysis. The effect indicator was the odds ratio (OR) value and its corresponding 95% confidence interval (CI) [20]. Additonally, the pooled ORs of LIPC genotypes of rs2070895 and rs1800588 polymorphisms in all genetic inheritance models (allele, additive, recessive, and dominant models) were respectively calculated to analyze their relationships with the risk of hypertension. The heterogeneity test is based on the Q test and I^2^ statistics [21]. If the heterogeneity test showed significant results (*P* < 0.05 or I^2^ > 50%), the random effect model was used to calculate the combined effect value. Otherwise, the fixed effect model was selected to merge the data (*P* > 0.05 and I^2^ < 50%).

### Publication bias and sensitivity analysis

Egger’s test was used to evaluate publication bias [22]. Sensitivity analysis was performed by “leaving one out” for all models.

## Results

### General characteristics of the selected publications

**Fig. 1:**
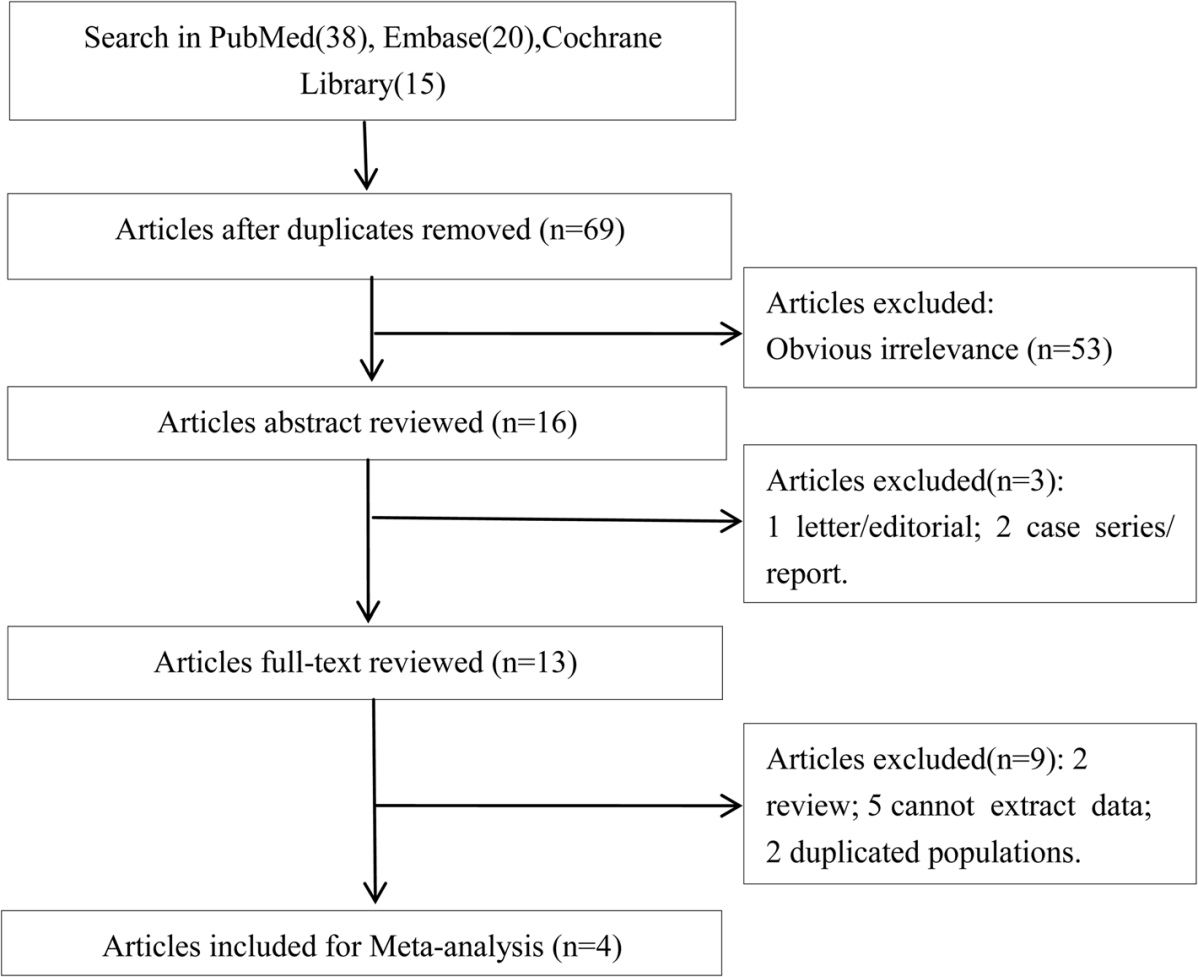
Flow chart shows the process of study selection

Using our search strategy, four related articles were identified [23–26]. The flow chart of the selection process of included publications is summarized in Fig. 1. A total of 83 studies were identified in PubMed (38), Embase (30) and Cochrane Library (15). After removing 14 repeated articles, 69 studies remainded. An additional 53 irrelevant articles were excluded, after which 16 studies remained. Subsequently, three studies were excluded after skimming the abstracts, including 1 letter and 2 case series or reports. Furthermore, 9 studies were excluded after reading the complete publication, which included 2 reviews, 5 unqualified data and 2 duplicated populations. As a result, four qualified studies were subjected to the meta-analysis (Fig. 1).

A total of 2599 participants were enrolled in the four included studies from 2007 to 2014. There were 1257 patients with hypertension in the case group, in which hypertensive disorder complicating pregnancy patients were included in studies by Bernard [23] and Lin [24], while 1342 healthy subjects were included as controls. The included studies were performed in Canada, Mexico, and China (Table 1). The main method used for SNP detection were PCR-ASO, PCR-RFLP and PCR-AIRS. There were no significant differences in demographic characteristics between the case and control groups of the included studies, with most patients being middle-aged and old, and of femlae gender. Analysis of the literature quality revelaed NOS scores is of 5–7 (Table 2), indicating a high quality. The results of HWE tests demonstrated that the genotypes distribution in controls was consistent with HWE in most included articles except for the control group of Yin’s study [26].

**Table 1:** The character of included literatures

Author	Public Year	Location	Detection method	SNP	NOS	Group	N	Age	Gender (Male/Female)	SNP			HWE	
										CC(GG)	CT(GA)	TT(AA)	Χ^2^^a^	P
Bernard N	2007	Canada	PCR-ASO	rs1800588	6	HDCP	106	27 ± 4	0/106	63	40	3	2.776	0.0957
						Control	169	27 ± 4	0/169	56	37	13		
Lin H	2014	China	PCR-RFLP	rs1800588	6	HDCP	321	29.1 ± 5.6	0/321	127	151	43	0.267	0.6055
						Control	331	27.9 ± 4.2	0/331	126	160	45		
				rs2070895		HDCP	321	29.1 ± 5.6	0/321	120	151	50	0.081	0.7754
						Control	331	27.9 ± 4.2	0/331	119	161	51		
Ríosgonzález BE	2014	Mexico	PCR-RFLP,PCR-AIRS	rs1800588	5	Case	160	47 ± 10	121/39	38	67	49	0.002	0.9691
						Control	160	47 ± 11	114/46	35	78	44		
Yin RX1	2012	China	PCR-RFLP	rs2070895	7	Case	330	49.9 ± 16.2	330/0	124	168	38	1.058	0.3036
						Control	338	48.8 ± 11.8	338/0	158	152	28		
Yin RX2	2012			rs2070895		Case	340	48.3 ± 15.8	0/340	143	131	66	4.716	0.0299
						Control	344	47.1 ± 11.2	0/344	162	160	22		

*SNP* Single Nucleotide Polymorphism, ^a^: likelihood-ratio Χ^2^; *NOS* Newcastle-Ottawa Scale, *N* The total number of including, *SNP* Single nucleotide polymorphisms, *HWE* Hardy-Weinberg equilibrium tests of control, *HDCP* Hypertensive disorders complicating pregnancy, 1:Male; 2: Female; *PCR-ASO* Polymerase chain reaction-allele-specific oligonucleotide, *PCR-RFLP* Polymerase chain reaction-restriction fragment length polymorphism, *PCR-AIRS* Polymerase chain reaction-artificial introduction of restriction sites

**Table 2:** The quality assessment of the included studies based on scores of Newcastle-Ottawa Scale

Author	Public Year	Representativeness of the cases	Case definition adequate	Ascertainment of exposure	Same method of ascertainment for cases and controls	Control for important factor or additional factor	Selection of controls	Definition of controls	Non-response rate	Total quality scores
Bernard N	2007	1	1	1	1	1	0	0	1	6
Lin H	2014	1	1	1	1	1	0	0	1	6
Ríosgonzález BE	2014	1	0	1	1	1	0	0	1	5
Yin RX	2012	1	1	1	1	0	1	1	1	7

### Quantitative data of meta-analysis

The present study was conducted to analyze the correlation of LIPC rs2070895 and rs1800588 polymorphisms with the susceptibility to hypertension in all genetic inheritance models. Initially, the heterogeneity test was performed. The appropriate effect model was used to calculate the combined effect value according to the *P* value and I^2^ statistics of Q test. The heterogeneity test revealed striking heterogeneity between studies associated with the LIPC rs1800588 polymorphism in additive and recessive models, as well as studies associated with the LIPC rs2070895 polymorphism in allele, additive and recessive models (*P* < 0.05, *I*^*2*^ > 50%). Therefore, the random effect model was used to calculate the OR value and 95% CI. A fixed effect model was used for data consolidation of other models of LIPC rs1800588 and rs2070895 polymorphisms because no obvious heterogeneity was found (*P* > 0.05, *I*^*2*^ < 50%). The merged results are presented in Table 2.

**Fig. 2:**
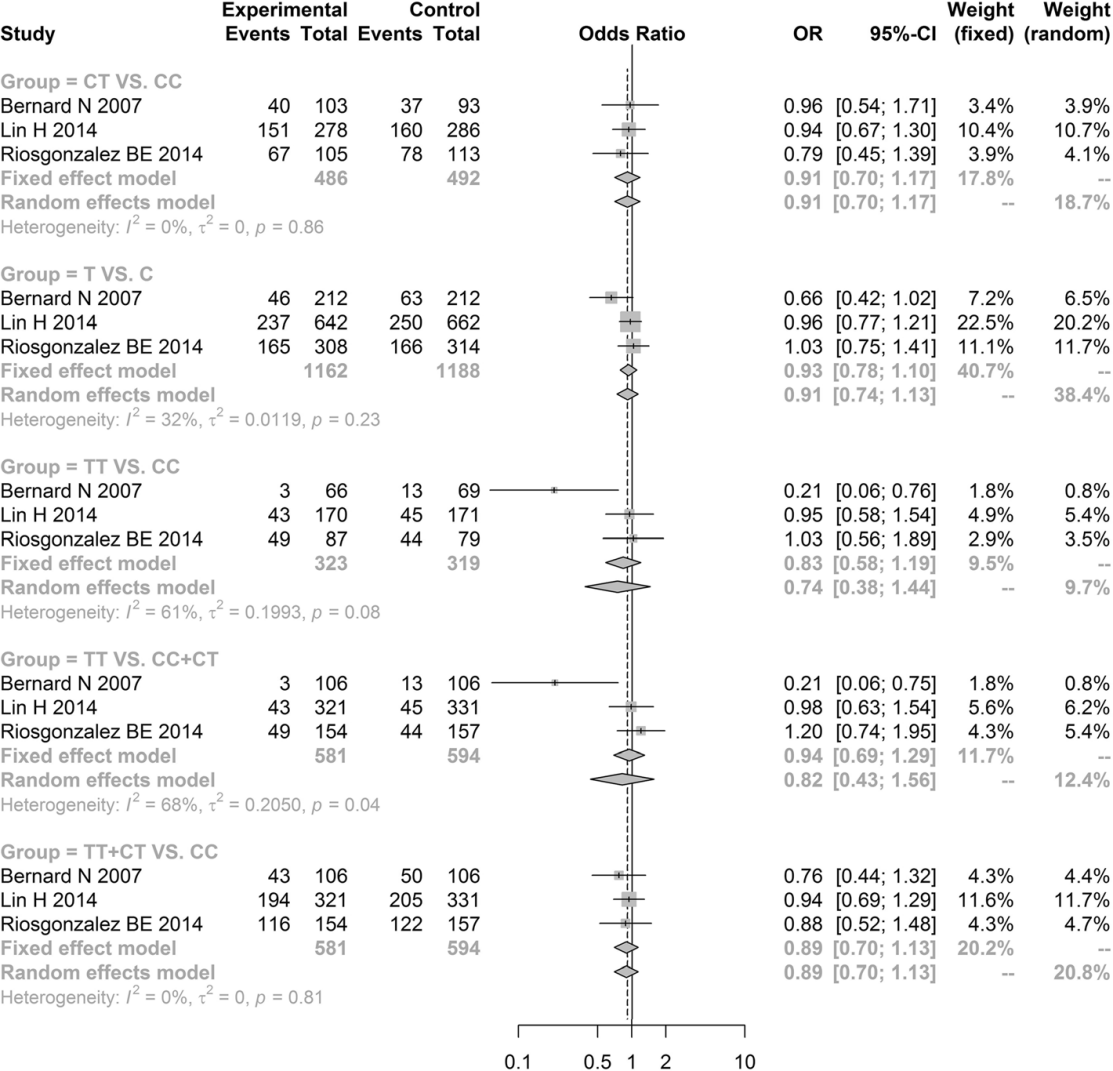
Forest plot of odd ratio obtained from the analyses of different LIPC rs1800588 polymorphism models of hypertension

**Fig. 3:**
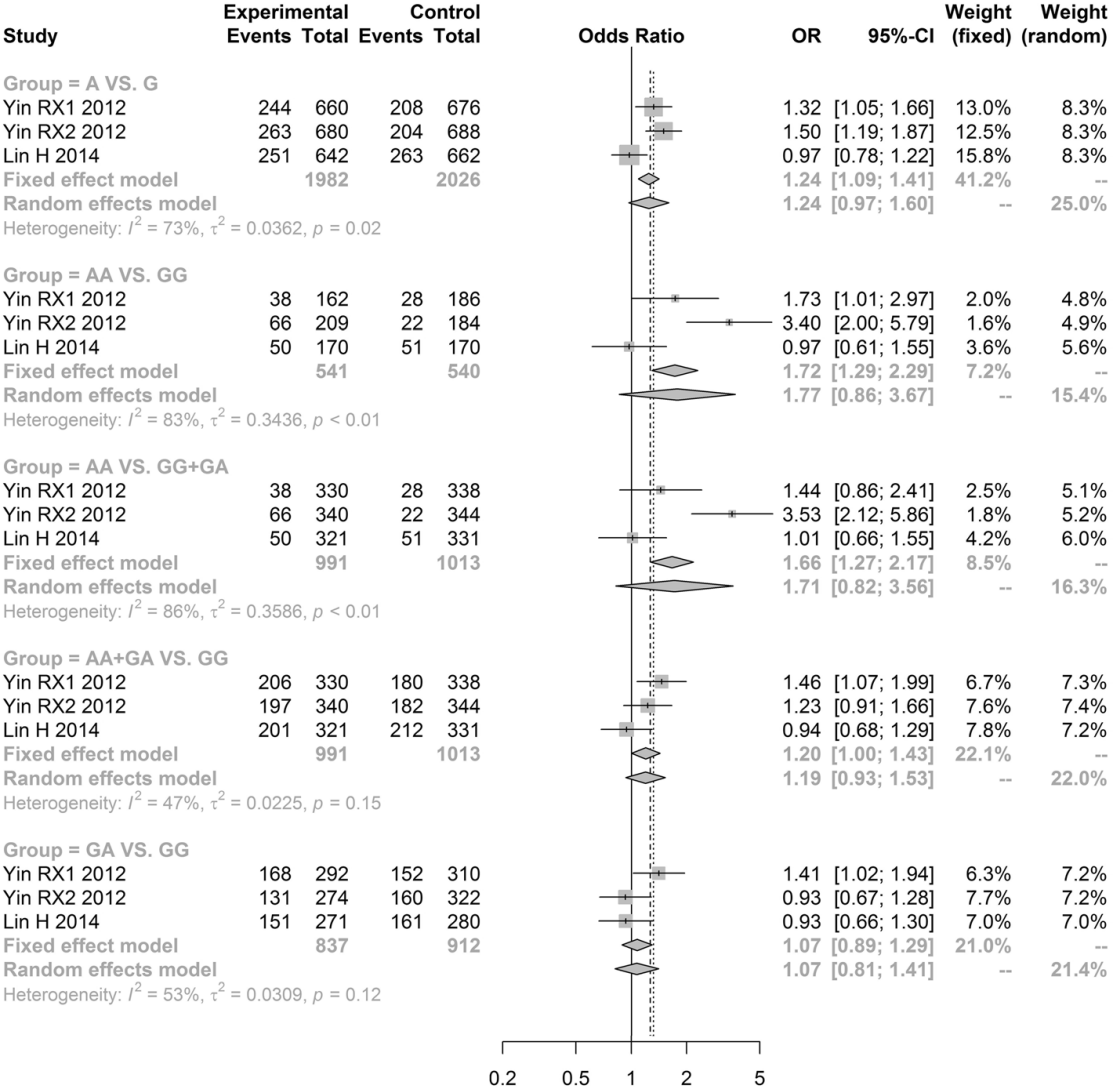
Forest plot of odd ratio obtained from the analyses of different LIPC rs2070895 polymorphism models of hypertension

The meta-analysis results revealed no significant difference in the LIPC rs1800588 polymorphism between the hypertension group and non-hypertension group under all genetic models [allele model (T vs. C, OR = 0.9276, 95% CI: 0.7838–1.0979); additive model (TT vs. CC, OR = 0.7446, 95% CI: 0.3839–1.4441) and (CT vs. CC, OR = 0.9090, 95% CI: 0.7038–1.1741); recessive model (TT vs. CC + CT, OR = 0.8176, 95% CI: 0.4286–1.5597); dominant model (TT + CT vs. CC, OR = 0.8882, 95% CI: 0.6974; 1.1312)] (Fig. 2, Table 3). Notably, our results showed that LIPC rs2070895 polymorphism were significantly related to an increased hypertension risk under the dominant model (AA+GA vs. GG, OR = 1.1954, 95% CI: 1.0001–1.4288; Fig. 3, Table 3). However, there was no significant difference in three other genetic models [allele model (A vs. G, OR = 1.2428, 95% CI: 0.9664; 1.5982); additive model (AA vs. GG, OR = 1.7723, 95% CI: 0.8570; 3.6651) and (GA vs. GG, OR = 1.0693, 95% CI: 0.8128; 1.4067); recessive model (AA vs. GG + GA, OR = 1.7115, 95% CI: 0.8225; 3.5612)] (Fig. 3, Table 3). Because the OR values in all models were larger than 1, gene A under the dominant model in LIPC rs2070895 polymorphism may be a dominant risk factor for the susceptibility to hypertension.

**Table 3:** The results of meta-analysis

SNP	Gene model	Sample size			Test of association			Model	Test of heterogeneity^a,b^			Publication bias^c^	
		K	Cases	Control	OR (95% CI)	Z	P		Q	*P*	*I*^*2*^ (%)	*t*	*P* value
rs1800588	T VS. C	3	1162	1188	0.9276 [0.7838; 1.0979]	0.87	0.3822	F	2.93	0.23	31.7	1.0914	0.4722
	CT VS. CC	3	486	492	0.9090 [0.7038; 1.1741]	0.73	0.4649	F	0.30	0.86	0.0	0.5385	0.6855
	TT VS. CC	3	323	319	0.7446 [0.3839; 1.4441]	0.87	0.3829	R	5.13	0.08	61.0	2.2042	0.2711
	TT VS. CC + CT	3	581	594	0.8176 [0.4286; 1.5597]	0.61	0.5412	R	6.26	0.04	68.1	2.9604	0.2074
	TT + CT VS. CC	3	581	594	0.8882 [0.6974; 1.1312]	0.96	0.3369	F	0.41	0.81	0.00	1.7230	0.3348
rs2070895	A VS. G	3	1982	2026	1.2428 [0.9664; 1.5982]	1.69	0.0903	R	7.51	0.02	73.4	0.5956	0.6580
	AA VS. GG	3	541	540	1.7723 [0.8570; 3.6651]	1.54	0.1226	R	12.07	< 0.01	83.4	0.1520	0.9040
	AA VS. GG + GA	3	991	1013	1.7115 [0.8225; 3.5612]	1.44	0.1506	R	13.92	< 0.01	85.6	0.1266	0.9198
	AA+GA VS. GG	3	991	1013	1.1954 [1.0001; 1.4288]	1.96	0.0498	F	3.80	0.15	47.4	1.0798	0.4756
	GA VS. GG	3	837	912	1.0693 [0.8128; 1.4067]	0.48	0.6319	R	4.22	0.12	52.6	0.7105	0.6068

*OR* Odds ratio, *CI* confidence interval, *K* The number of included studies, *R* Random, *F* Fixed

^a^Random-effects model was used when the P for heterogeneity test< 0.05, otherwise the fixed-effect model was used. ^b^*P* < 0.05 is considered statistically significant for Q statistics. ^c^Egger‘s test to evaluate publication bias, *P* < 0.05 is considered statistically significant

### Publication bias and sensitivity analysis

**Fig. 4:**
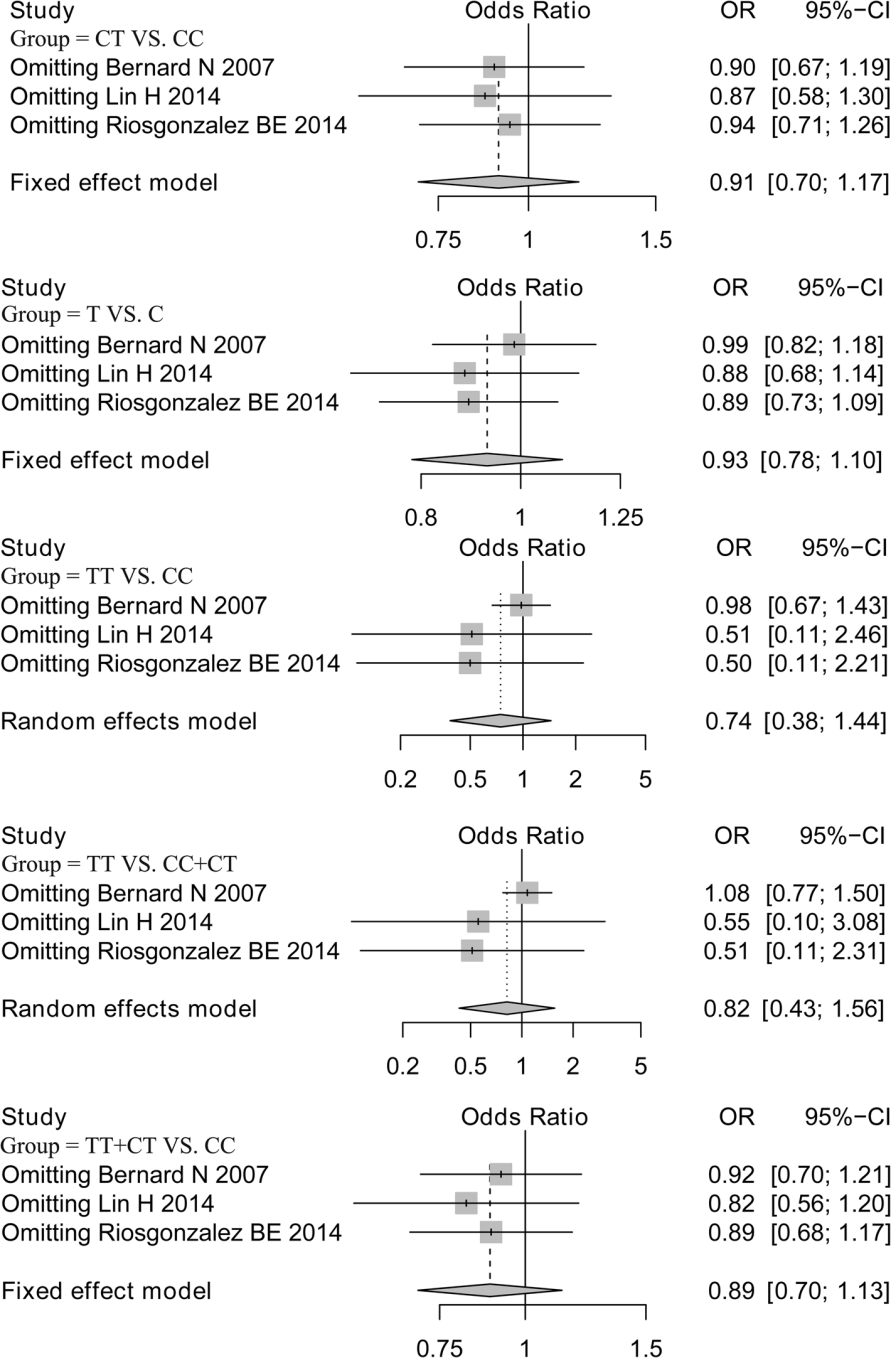
Sensitivity analysis of LIPC rs1800588 polymorphism after omitting each study in all genetic models

**Fig. 5:**
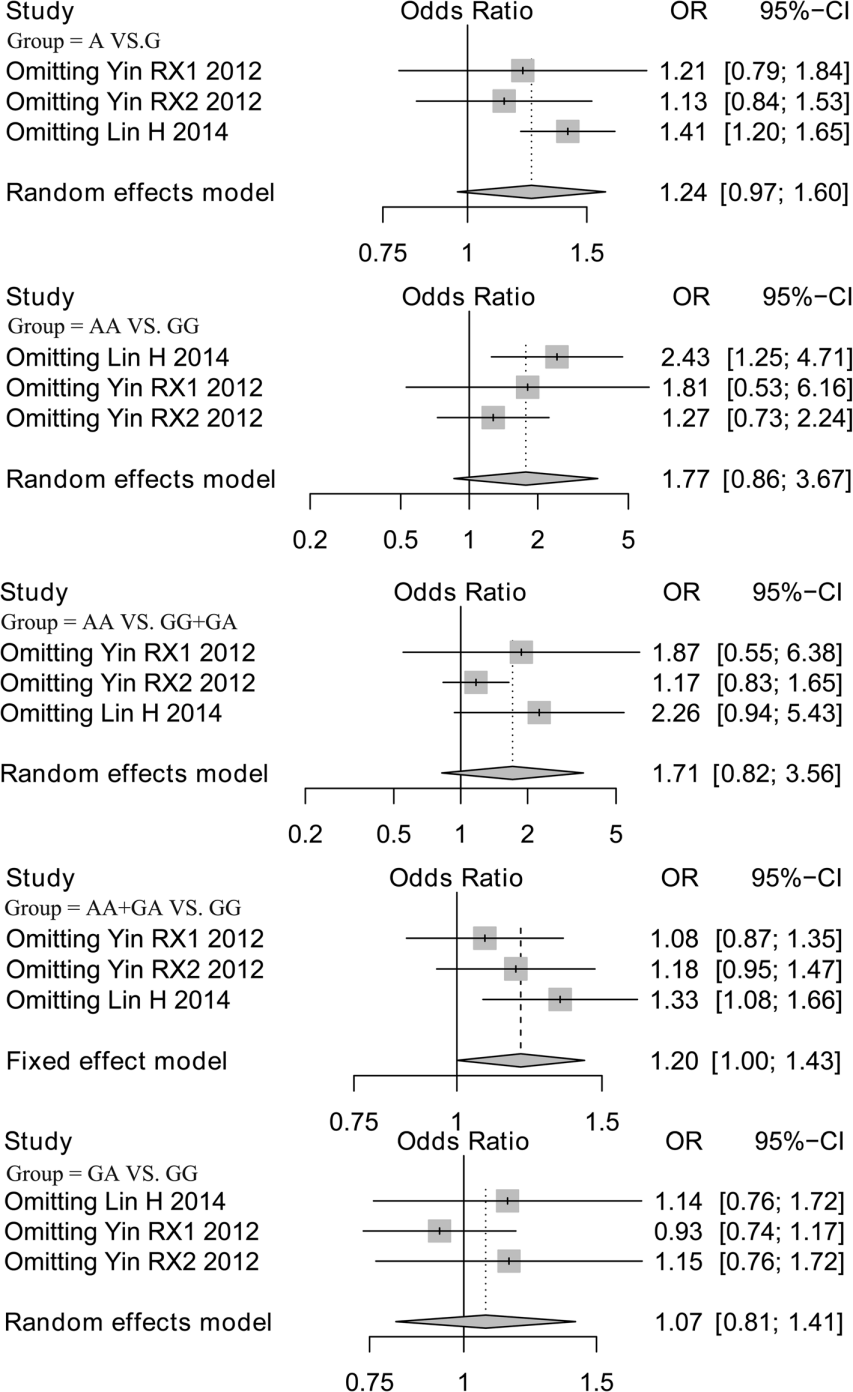
Sensitivity analysis of LIPC rs2070895 polymorphism after omitting each study in all genetic models

Analysis of publication bias demonstrated that the Egger’s test of LIPC rs1800588 and rs2070895 polymorphisms showed no publication bias, supporting the reliability of our results (Table 3). Sensitivity analysis was performed on all models by omitting each study. The results for the LIPC rs1800588 polymorphism were similar in all genetics models, indicating that the results were stable (Fig. 4). However, after eliminating the study by Lin [24], the results for the LIPC rs2070895 polymorphism under allele, additive (AA VS. GG) and dominant models were changed, and no changes was found in other models (Fig. 5).

## Discussion

In this meta-analysis, we pooled related studies of the frequency distribution of LIPC -250G/A (rs2070895) and -514C/T (rs1800588) polymorphisms in hypertension patients and normal individuals to assess the relationship of LIPC gene polymorphisms and hypertension risk. The LIPC rs1800588 polymorphism did not appear to be associated with an increased risk of hypertension. Notably, the LIPC rs2070895 polymorphism frequencies were significantly increased in the hypertension group compared to in controls under the dominant model, and OR values of LIPC rs2070895 polymorphism under all models were larger than 1. Therefore, the LIPC rs2070895 polymorphism may be a risk factor for the susceptibility to hypertension.

The human HL protein encoded by the LIPC gene has phospholipase A1 and triglyceride hydrolytic enzyme activities, and plays an important role in plasma lipid transport [27]. It is well known that LIPC is independently involved in the reconstruction of high-density lipoprotein cholesterol (HDL) and metabolism of low density lipoprotein (LDL) [28]. Conthe et al. demonstrated that in patients with hypertension, the serum level of HDL-C was decreased [29]. Similarly, a lower serum HDL level may be a prognosis factor for patients with cardiovascular risk among essential hypertension patients [30]. In contrast, an increased LDL level is known to be associated with the new-onset and development of hypertension [31]. In general, dyslipidemia is significant risk factors for hypertension, which precedes the occurrence of clinical hypertension [32]. Thus, LIPC polymorphisms may be closely associated with increased risk of hypertension by regulating HDL, LDL, and other lipid metabolism levels.

Some studies showed a strong connection between the LIPC -250G/A (rs2070895) polymorphism and lipoprotein metabolism disease, such as hyperlipidemia [33, 34] and diabetes [8]. Additionally, differences in the HDL-C and LDL-C between Han and Bai Ku Yao populations may be related to the LIPC rs2070895 polymorphism under the dominant model (AA+GA vs. GG) [35]. Interestingly, our study revealed that the LIPC rs2070895 polymorphism was strongly related to an increased hypertension risk under the dominant model (AA+GA vs. GG).

The T allele in LIPC -514C/T polymorphism has been shown to be related to higher plasma HDL-C levels [36], and the T allele may decrease the susceptibility to nonalcoholic fatty liver disease [37]. Although one included study by Bernard et al. showed that TT homozygotes at LIPC -514 had a decreased risk of gestational hypertension compared to CC homozygotes [23], our results showed that the LIPC -514C/T (rs1800588) polymorphism was not associated with hypertension under all genetic models. Similarly, not all studies found an association between the LIPC -514C/T polymorphism and lipoprotein metabolism. A study of Iranian population revealed no difference in the frequency of the T allele between coronary heart disease and normal arteries [38]. Additionally, the C and T alleles frequencies of the -514C/T polymorphism showed no significant difference between coronary artery disease and normal groups [8, 39, 40]. These differences may be realted to differences in sample size, lifestyles, and gender of the study populations.

Heterogeneity test results revealed a significant difference between included studies. The potential heterogeneity sources included different regional living habits, living environments, and the economic development levels. Additionally, the effects of other confounding factors such as gender and age can lead to high heterogeneity.

There were some limitations in this research. First, because of incomplete data and the small sample sizes of the included studies, covariate correction and further subgroup analysis were not performed, although these factors may have confounded the results of the meta-analysis. Second, the population was not representative, as the population in Yin’s study (female) did not fit HWE. Third, the results of sensitivity analysis regarding the LIPC rs2070895 polymorphism in the allele, additive, and dominant models were unstable after elimination of Lin’s study, which requires further analysis. Fourth, hypertensive disorders complicating pregnancy may have affected our results.

In summary, the present study showed that the frequencies of the AA+GA genotypes of the LIPC rs2070895 polymorphism are related to an increased risk of hypertension. However, the LIPC rs1800588 polymorphism was not associated with the susceptibility to hypertension. Because the related studies and sample size were limited, the conclusions of this study should be verified by high-quality studies with larger sample size.

## Abbreviations

AHRQ: Agency for healthcare research and quality
CI: Confidence interval
HDL: High-density lipoprotein cholesterol
HDPC: Hypertensive disorder complicating pregnancy
HWE: Hardy-Weinberg equilibrium
LDL: Low density lipoprotein
LIPC: Hepatic lipase gene
NOS: Newcastle-Ottawa Scale
OR: Odds ratio
PICO: Participants, interventions, comparison and outcome measures
PRISMA: Preferred Reporting Items for Systematic Reviews and Meta-Analyses
SNPs): Single nucleotide polymorphisms
